# Mineral and organic growing media have distinct community structure, stability and functionality in soilless culture systems

**DOI:** 10.1038/srep18837

**Published:** 2016-01-05

**Authors:** Oliver Grunert, Emma Hernandez-Sanabria, Ramiro Vilchez-Vargas, Ruy Jauregui, Dietmar H. Pieper, Maaike Perneel, Marie-Christine Van Labeke, Dirk Reheul, Nico Boon

**Affiliations:** 1 Laboratory of Microbial Ecology and Technology (LabMET), Ghent University, Coupure Links 653, B-9000 Gent, Belgium; 2 Department of Plant Production, Ghent University, Coupure Links 653, B-9000 Gent, Belgium; 3 Peltracom NV, Skaldenstraat 7a, B-9042 Ghent-Desteldonk, Belgium; 4 Microbial Interactions and Processes Research Group, Department of Molecular Infection Biology, Helmholtz Centre for Infection Research, Inhoffenstraße 7, D-38124, Braunschweig, Germany

## Abstract

The choice of soilless growing medium for plant nutrition, growth and support is crucial for improving the eco-sustainability of the production in horticultural systems. As our current understanding of the functional microbial communities inhabiting this ecosystem is still limited, we examined the microbial community development of the two most important growing media (organic and mineral) used in open soilless horticultural systems. We aimed to identify factors that influence community composition over time, and to compare the distribution of individual taxa across growing media, and their potential functionality. High throughput sequencing analysis revealed a distinctive and stable microbial community in the organic growing medium. Humidity, pH, nitrate-N, ammonium-N and conductivity were uncovered as the main factors associated with the resident bacterial communities. Ammonium-N was correlated with *Rhizobiaceae* abundance, while potential competitive interactions among both *Methylophilaceae* and *Actinobacteridae* with *Rhizobiaceae* were suggested. Our results revealed that soilless growing media are unique niches for diverse bacterial communities with temporal functional stability, which may possibly impact the resistance to external forces. These differences in communities can be used to develop strategies to move towards a sustainable horticulture with increased productivity and quality.

In the U.S., Canada and Europe, 95% of greenhouse vegetables, particularly tomatoes, are produced in soilless greenhouse plant cultivation systems using horticultural growing media^1^. Open soilless horticultural systems have advantages over traditional systems in that the nutrients, oxygen and water required for a healthy plant growth are controlled^2^ and that soil-borne pathogens can be circumvented^3^,^4^. In Western Europe, nearly all greenhouse-grown tomatoes are produced on mineral growing medium comprised of inorganic synthetic fibres^5^. Mineral growing media are produced from diabase, limestone and cokes, which are melted together at 1500 °C and spun into fibres^6^. In contrast, peat and coconut are the most utilised organic-derived constituents of growing media produced in the EU^7^. While mineral growing medium has a neutral pH, high air content and low density, organic growing medium is characterised by its high organic matter content and capacity for cation exchange with the water solution irrigating the growing medium. In spite of these differences, the yield and number of tomato fruits (*Solanum lycopersicum*) was comparable among plants grown on either mineral or organic growing media over several consecutive years^8^.

The sustainability of soilless greenhouse systems relies heavily on increased yields and on the general efficacy of the growing process^4^. Disinfection measures are taken at the greenhouse to guarantee final yield and quality^4^. However, this results in the elimination of not only deleterious microorganisms but also of potentially beneficial microbial taxa for the plant. This may ultimately prevent the community from reaching equilibrium and stability, making these soilless cultivation systems at risk of successful pathogen invasion^4^. Biodiversity protects ecosystems against declines in their functionality, as a consequence of the functional redundancy through the co-existence of multiple species^9^,^10^. This can also lead to increased productivity^11^, due to positive impact on bacterial respiration, microbial biomass production, and plant nutrient storage. In addition, increased temporal functional stability and resistance to external forces, such as nutrient perturbations and invasive species, have been reported^10^,^12^.

The complex plant-associated microbial community, also referred to as the “second genome of the plant”, is crucial for plant health, growth and development^13^. Previous work investigating microbial communities associated with growing media has mainly focused on the absence of pathogenic bacteria and fungi^14^. There is limited understanding of the factors that influence community composition over time, the distribution of individual taxa across growing media, and their potential functionality. The lack of effective control strategies aiming at enhancing productivity^15^ increases our need to closely monitor the rhizosphere, the growing medium and its microbial populations.

In this study, we examined the microbial community development of the two most important growing media used in open soilless horticultural systems: organic (GB) and mineral (RW). We hypothesised that RW and GB growing media develop different community structure with potentially unique functionalities. Much of the fluctuation within the rhizosphere is defined by the plant root and the conditions in the growing medium. Therefore, the rhizosphere and the microbial community associated with the growing medium are strongly connected. Knowledge regarding these differences can be used to develop strategies towards sustainable horticulture, with enhanced productivity and quality, and potentially increased resistance to external forces^12^. The hairy roots syndrome caused by *Agrobacterium rhizogenes* infection is a major issue in greenhouse horticulture, because total yield may decrease up to 10% in tomato plants^16^. In our study, naturally occurring *A. rhizogenes* infection was detected in some plants growing in the RW medium (RWS). The interactions occurring between the roots, rhizosphere and the growing medium and the potential resistance to external forces, represented in our study by *A. rhizogenes*, were also assessed.

## Results

### Identification of the microbial community associated with the growing medium

**Figure 1:**
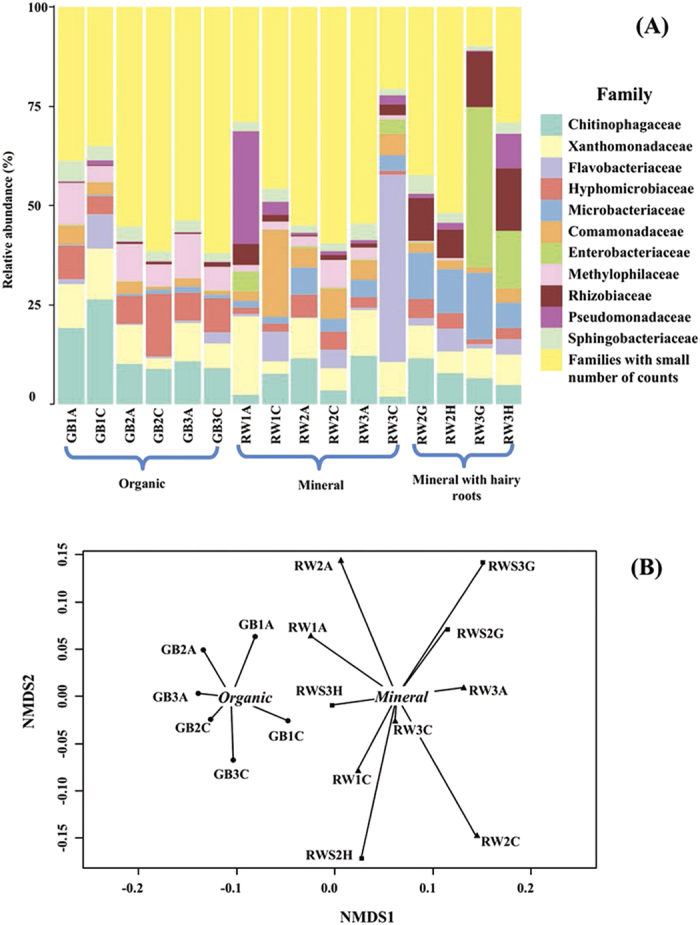
(**A**) Relative abundances of the bacterial families present in horticultural growing media. Families with the highest sequence count and their corresponding RDP classification are indicated. RW: mineral growing medium; GB: organic growing medium, RWS: mineral medium with hairy roots. Dataset was rarefied to the lowest sequence count; relative abundances were calculated summing the counts of OTUs belonging to the same family. (**B**) Community structure was significantly different between growing media types. Analysis of multivariate homogeneity of group dispersions (variances) was performed and non-metric multidimensional scaling analysis was used to assess the similarity among bacterial communities. Symbols indicate the growing medium type: circles, organic growing medium (GB); triangles, mineral growing medium (RW); squares, mineral growing medium with hairy roots (RWS). The number in the legend specifies the time point and the letter refers to the sample replicate. For instance, “GB1A” refers to the replicate “A” of organic growing medium, collected at the first time point.

*Chitinophagaceae, Xanthomonadaceae, Flavobacteriaceae, Hypomicrobiaceae, Microbacteriaceae, Comamonadaceae, Enterobacteriaceae, Methylophilaceae, Rhizobiaceae, Pseudomonadaceae,* and *Sphingobacteriaceae* were the bacterial families with highest relative abundances in both growing media (Fig. 1A). *Chitinophagaceae, Methylophilaceae* and *Hypomicrobiaceae* were abundant in GB, while *Microbacteriaceae* were increased in RW. *Enterobacteriaceae, Verrucomicrobiaceae* and *Rhizobiaceae* were abundant in RWS and decreased in GB (Tables 1 and 2). Permutational multivariate analysis of variance (PERMANOVA) confirmed that growing media type, but not time point, significantly contributed to the differences in the relative abundances of bacterial families (*P* < 0.05, Fig. 1B). Analysis of the DGGE profiles showed that RWS samples grouped together regardless of time, while the rest of the samples tended to cluster according to time point (Supplementary Fig. 1). The total number of species was higher in GB (*P* < 0.05), while diversity and evenness among GB and RW was significantly different across time points (*P* < 0.05, Supplementary Table 1). RW and RWS showed consistent similarities in their diversity and evenness metrics (Supplementary Table 2).

**Figure 2:**
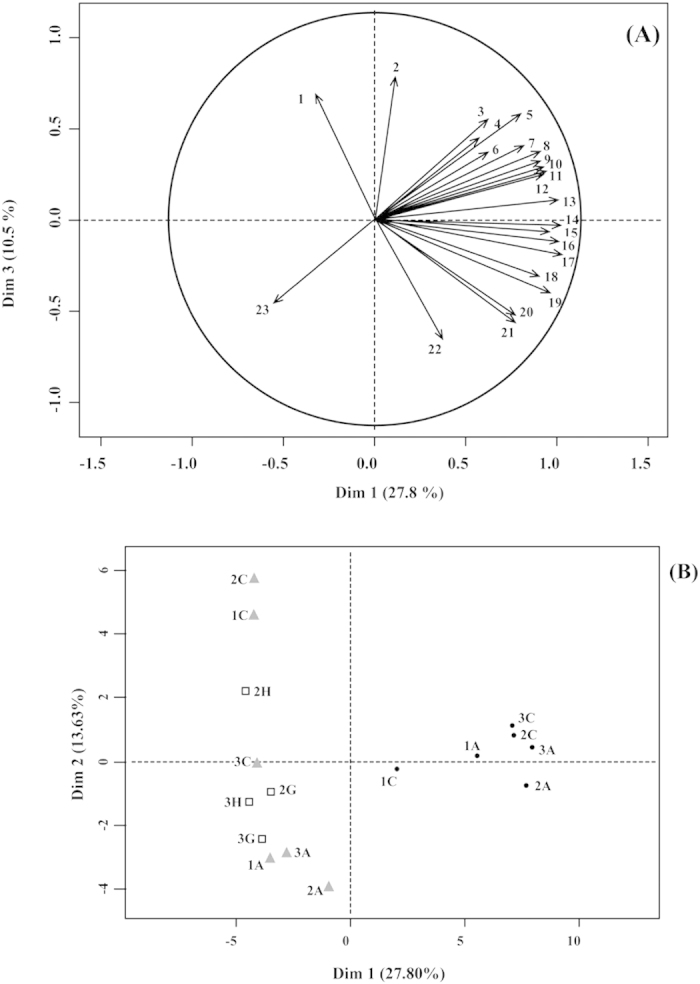
(**A**) Variations in the abundance of bacterial families in horticultural growing media. According to the correlation circle, the families belonging to the first component of the Multiple Factor Analysis (Dim. 1) are negatively correlated to the abundance of species belonging to *Rhizobiaceae* (23). Since there are more families in Dim 3., their contribution to the overall variance among samples is smaller. Dimension 3 described the families that were significantly correlated with RW (*P* < 0.05). 1, *Propionibacterineae*; 2, *Pseudonocardineae*; 3, *Rhodobacteraceae*; 4, *Caedibacter*; 5, Incertae *Rhizobiales*; 6, Unclassified *Nitrospira*; 7, *Methylophilaceae*; 8, *Gaiellaceae*; 9, *Acetobacteraceae*; 10, *Actinobacteridae*; 11, *Xanthobacteraceae*; 12, *Hahellaceae*; 13, *Sinobacteraceae*; 14, *Desulfobacteraceae*; 15, *Hyphomicrobiaceae*; 16, *Opitutaceae*; 17, *Gemmatimonadaceae*; 18, *Methylocystaceae*; 19, *Sorangiineae*; 20, *Hyophomonadaceae*; 21, *Chromatiaceae*; 22, *Rhodocyclaceae*; 23, *Rhizobiaceae*. (**B**) Multiple Factor Analysis map indicated that samples from organic growing medium (GB) displayed similar abundances across time points and differed from those in mineral medium (RW). Bacterial family abundances in samples of RW with hairy roots (RWS) were similar to those in RW. Symbols indicate the growing medium type: black circles for GB, grey triangles for RW and white squares for RWS. The number in the legend specifies the time point and the letter refers to the sample replicate. For instance, the circle labelled as “1A” refers to the replicate “A” of GB, collected at the first time point.

Multiple Factor Analysis (MFA) showed that families associated with GB were represented in Dimension 1 (*P* < 0.0001, Fig. 2A), accounting for 28% of the variance in relative abundances among all the samples. *Gemmatimonadaceae, Sinobacteraceae, Sorangiineae, Opitutaceae, Desulfobacteraceae, Actinobacteridae, Hahellaceae, Gaiellaceae, Hypomicrobiaceae, Methylophilaceae, Acetobacteraceae, Methylocystaceae, Conexibacteriaceae, Xanthobacteraceae* and Unclassified *Nitrospira* were significantly associated with GB. Bacterial families that correlated with RW were represented in Dim. 3 (*P* < 0.05) and explained 11% of the total variance. *Pseudonocardineae, Propionibacterineae, Bacteroidaceae, Commamonadaceae*, Incertae *Rhizobiales* and *Cryomorphaceae* were associated with this dimension. *Rhizobiaceae, Verrucomicrobiaceae, Planctomycetaceae, Simkaniaceae, Piscirickettsiaceae*, and *Caldilineaceae* were families associated with RWS and included in Dim. 5 (*P* < 0.05, Supplementary Table 3).

Tukey’s test for pairwise comparisons of group mean dispersions was performed using the vegan package in R. As demonstrated by the diversity and evenness measures (Supplementary Tables 1 and 2), the interaction between time and growing medium type was significant at the third time point (*P* < 0.05). Based on the relative abundances of the bacterial families and on the measures of alpha diversity and evenness, we validated the presence of distinctive and stable microbial communities associated with each growing medium (Tables 1 and 2 and Fig. 2B).

### Physicochemical and biological environments are unique between the different growing media

**Figure 3:**
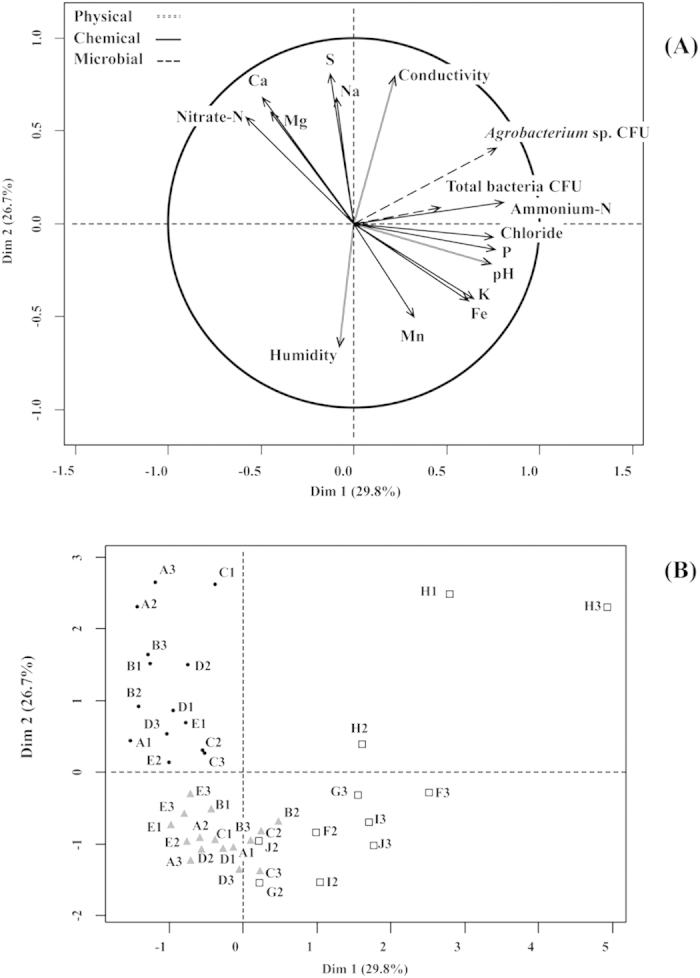
Physical and chemical characteristics of the growing media are unique for each environment. (**A**) Multiple Factor analysis of the physical and chemical characteristics of horticultural growing media. Correlation circle indicates the contribution of the variables driving the differences among growing media. Long vectors in the same direction indicate positive correlations among variables, whereas long vectors in the opposite direction indicate negative ones. (**B**) Multiple Factor Analysis highlighted the similarities among growing media samples over time, based on their physical and chemical features. Symbols indicate the growing medium type: black circles for GB, grey triangles for RW and white squares for RWS. The number in the legend specifies the time point and the letter refers to the sample replicate. For instance, the circle labelled as “1A” refers to the replicate “A” of GB, collected at the first time point.

Plant yield was determined at the end of the growing season for both the organic and mineral growing medium, and resulted in total accumulated yield (fresh weight) of 59.27 ± 1.52 kg.m^−2^ and 61.59 ± 0.86 kg.m^−2^ respectively. Calcium, magnesium, sulphate, nitrate-N, sodium and conductivity were higher in GB than Calcium, magnesium, sulphate, nitrate-N, sodium and conductivity were higher in GB than in RW (P < 0.05). in RW (*P* < 0.05), while ammonium-N, potassium, iron, and manganese were significantly higher in RW in comparison with GB (*P* < 0.05, Supplementary Table 4). Ammonium-N, pH, conductivity, potassium, sodium, iron and chloride were the highest in RWS (*P* < 0.05, Supplementary Table 5). Positive correlations between conductivity and nitrate-N were consistently detected in GB, while ammonium-N was associated with total CFU only at the third time point (*P* < 0.05, Table 3). In RW, pH was positively correlated with the *Agrobacterium* sp. CFU, while conductivity, ammonium-N, sulphates and sodium were negatively correlated with the total CFU. Only sulphates were positively correlated with sodium across time points (*P* < 0.05, Table 4). Positive correlations between calcium and *Agrobacterium* sp. and total CFU were found in RWS at all times (*P* < 0.05, Table 5). In contrast, humidity was negatively correlated with *Agrobacterium* sp. and total CFU when the hairy roots were first detected (*P* < 0.05). The goal of the MFA was twofold: to discriminate growing media based on the measured variables and to uncover the correlations among the physicochemical and biological characteristics within growing medium. In general, MFA showed that total bacteria, *Agrobacterium* sp. CFU, humidity, pH, sulphate and conductivity were the traits with highest contribution to the total variance among samples (Fig. 3A). Ammonium-N and *Agrobacterium* sp. CFU were the two variables with the highest correlation to Dimension 1 (*P* < 0.0001, Supplementary Table 6), which accounted for 29.8% of the variance. The square correlation ratios measure the degree of association between variables and a particular axis. Thus, the cos^2^ between the coordinates of the samples and growing medium type revealed that the above were the main characteristics describing the RWS medium on Dim. 1 (cos^2^ > 0.5). Dim. 2 (26.7% of the total variance) was constructed by the features of GB (sulphate, conductivity, sodium, magnesium, calcium and nitrate-N), while potassium, manganese, iron and humidity were included in Dim. 3 with RW (*P* < 0.05, 13.6% of the variance). We confirmed that each growing medium was characterised by a unique set of physicochemical and biological variables, which is preserved over time (Fig. 3B).

### Correlation of bacterial families with physicochemical and biological characteristics

**Figure 4:**
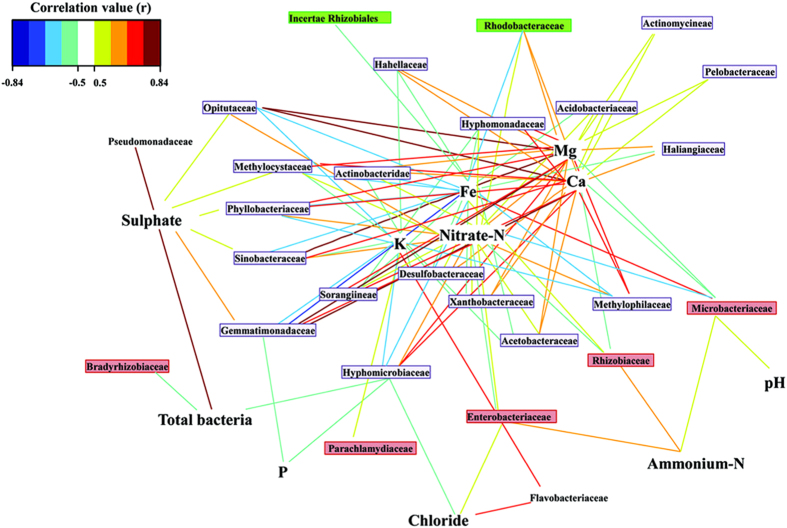
Network graph based on the regularised canonical correlations between bacterial family abundance and physicochemical characteristics of growing medium. Correlations (r) have been filtered for an absolute correlation above 0.5 and are coloured following the key shown. According to the graphing algorithm, stronger correlations are shorter lines, and families with similar abundances within growing medium tend to cluster closely. This representation reveals the relationship between clusters of families linked to the different physical and chemical characteristics of the environment, thus potentially uncovering growing medium-specific populations. In green, bacterial families correlated with RW; in red, bacterial families associated with RWS, in purple, families correlated with GB.

Instead of computing the Pearson correlation coefficients between each pair of variables, bipartite networks were inferred using a pair-wise similarity matrix obtained from the Regularised Canonical Correlation Analysis (Fig. 4). The values in the similarity matrix were computed as the correlation between the relative abundances of bacterial families and the characteristics of the growing medium projected onto the space spanned by the first components retained in the analysis. Three relevant components were obtained setting a threshold to 0.5. In this way, ammonium-N was correlated with *Rhizobiaceae* abundance. Families associated with RW were correlated with iron (*P* < 0.05), while potassium, magnesium, calcium and nitrates were associated with GB (*P* < 0.05). Correspondence Analysis (CA, Supplementary Fig. 2) was used to reveal the association between growing medium (rows) and physicochemical variables and relative abundances (columns). Chi-square statistic indicated strong link between growing medium and both physicochemical variables and relative abundances (*P* < 0.05). The coordinates of row/column variables represent the number of standard deviations the row/column variables are away from the barycentre^17^. Therefore, the highest coordinates in Dim.1 belonged to *Agrobacterium* sp. CFU, *Solimonadaceae*, Ammonia-N and P (rows) and RWS and time point 3 (columns), all of which explained 92.9% of the variance. Dim. 2 was driven by the relationships among the most abundant families detected in GB at time point 2. The observed interactions confirmed the results of previous correlation analyses (Tables 1 and 2).

### Estimation of the matric potential of the growing media

The water retention curve of the two growing media was determined. Saturated volumetric water content of RW (θ_s_ = 0.9834) agreed with previous reports of Wallach^18^, while the saturated volumetric water content of GB was θ_s_ = 0.935. The volumetric water content (θ_v_ = 85.3%) of RW corresponds to a matric potential of −0.6 kPa, whereas θ_v_ = 83.1% for RWS corresponds to a matric potential of −0.61 kPa. For the organic growing medium, θ_v_ = 81.93% represents a matric potential of −0.46 kPa.

### Potential resistance to external forces differed between growing media

Seven RW samples (46.7% out of fifteen) were positive for *A. rhizogenes* biovar 2, whereas samples from GB were negative for any of the tested species of *Agrobacterium* sp. (Supplementary Table 7). Phytopathogenic *Agrobacterium* sp. strains harbour the genes required for T-DNA process and transfer in the virulence regions (*virC*) of the root inducing (pRi) plasmids^19^,^20^. Therefore, RW samples positive for *A. rhizogenes* biovar 2 and all RWS samples were screened for the presence of the *virC* pathogenicity gene, and all RWS tested positive. Plate counts on the selective medium confirmed the results of the multiplex PCR. Results of the colony PCR in randomly selected wild type isolates validated the presence of the *virC* gene and of *A. rhizogenes* biovar 2 in RWS (Supplementary Table 7). Differences in *Agrobacterium.* sp. CFU between growing media were influenced by time and were lower in RW when compared with RWS (*P* < 0.05, Supplementary Table 7).

## Discussion

Based on the relative abundances of the families associated with each growing medium, we validated the presence of a competitive, distinctive and stable microbial community structure in the organic growing medium. Further, we identified humidity, potassium content, pH and conductivity as the main physicochemical characteristics driving microbial communities in the growing medium.

High-throughput sequencing combined with molecular techniques uncovered the structure of the growing medium-associated microbiota. GB harboured higher bacterial diversity than RW and RWS. Further, GB displayed similar abundances of bacterial families across time points, while both RW and RWS displayed larger variability. These differences could be associated with the different structure and composition of the two types of growing medium, which may provide unique niches for the microbial community^21^. The density and the biodiversity of the microbial community may be affected by the age of growing medium^22^. Biodiversity in soilless systems with mineral growing media is low at the start of a crop^4^, then it increases within weeks^23^, reaching stability after six weeks of plant growth^24^. As described in previous reports^21^, our uncultivated RW medium showed low amounts of nutrients and total bacteria CFU (<10^2^ CFU g^−1^) in comparison with GB (2.2 × 10^7^ CFU g^−1^). Quantification of viable cell counts (CFU) from the culturable aerobic microflora colonizing different parts of the system, such as root zone, nutrient solution, growing media and system devices (tubes, gutters, etc.), has been performed^25^. However, only 1 to 10% of the microflora may be recovered from techniques based on a plate culturing on R2A agar, providing limited information about the entire community present on each growing medium. For this reason, we complemented cultivation studies with molecular characterization of the microbial communities.

The increase in microbial biodiversity observed in the growing medium can be attributed to plant activity. Plants exude up to 21% of their photosynthetically fixed carbon into the root-soil interface^26^, feeding the microbial communities and influencing their activity and diversity^14^. Berendsen, *et al.*^27^ suggested that plant species can select bacteria through the production of specific root exudates and hence shape the microbiome of the plant. We used eggplants grafted on a tomato root stock known for its high exudation capacity (*Solanum lycopersicum* L. x *Solanum habrochaites*). The root exudation in both growing media was estimated to be similar, because cultivated eggplants showed comparable growth characteristics and yield. We found that even after six months, the microbial community in the mineral growing medium (both in RW and RWS) showed high variability across time points. Garbeva, *et al.*^28^ hypothesised that in a stable system, each microhabitat is occupied by organisms capable of colonizing niches. A diverse and stable ecosystem at the microhabitat level will resist environmental stresses^29^ and potentially, pathogen invasion. Mendes, *et al.*^14^ suggested that the relative abundance of several bacterial taxa may be an indicator of disease suppression. In this way, increased resistance to pathogen invasion may be related to the total microbial biomass in the growing medium, which competes with pathogens for resources or may cause inhibition through direct antagonism^15^. Mendes, *et al.*^14^ identified Actinobacteria, γ- and β-Proteobacteria (*Pseudomonadaceae, Burkholderiaceae, Xanthomonadales*) and Firmicutes (*Lactobacillaceae*) as the most dynamic taxa associated with disease suppression in natural soil. In our study, *Rhodocyclaceae* and *Methylophilaceae* (β-Proteobacteria), were correlated to GB, as well as other α-, β and γ-Proteobacteria, such as *Hyphomicrobiaceae, Xanthobacteraceae, Phyllobacteriaceae*, and *Chromatiaceae*. Actinobacteria such as *Gaiellaceae* and *Conexibacteraceae* were also positively correlated with GB. Furthermore, the abundance of *Rhizobiaceae* (such as *Agrobacterium* sp.) was negatively correlated to the abundances of *Methylophilaceae* and *Saprospiraceae* in GB. Thus, the relative abundance of several taxa and the stability of a microbial community may be related to the resistance against external invaders, supporting the theory of general suppression. Even though *Agrobacterium* sp. was detected in both growing media (on average 7.6 × 10^3^ CFU/ml in GB, 2.4 × 10^4^ in RW and 1.0 × 10^6^ CFU/ml in RWS, across time points), samples from GB were negative for the presence of the *virC* pathogenicity gene. Neither the total CFU nor the presence of particular microbial taxa have been directly associated with resistance to *Agrobacterium rhizogenes*^27^.

The plant, as well as the complex biological, chemical and physical interactions in the growing medium influence the microbial communities of the rhizosphere. Previous reports identified pH as the main driver of microbial communities in soil^30^. Soil moisture is often associated with pH and may have impacted the community composition among GB, RW and RWS. Moreover, GB showed higher relative abundances of *Actinobacteridae* and α-Proteobacteria, which have been associated with soil pH^30^. The mobility of rhizobacteria may be limited by the humidity in the growing medium, where water-filled pores may be as large as the bacterial cells^31^. Although *Agrobacterium* sp. was detected in GB and RW, the hairy roots were not visually present in GB. Our results indicate that differences in pore size and water distribution between GB and RW may have impacted the mobility of *Agrobacterium* sp., resulting in decreased disease incidence. High yields of hairy roots indicating *A. rhizogenes* invasion have been observed when the nitrate-ammonium (NO_3_^−^-N/NH_4_^+^) ratio was close to 5 with 115 mg NH_4_^+^-N/l of soil and 553 mg NO_3_-^−^N/l of soil^32^. In our test, the NO_3_^−^-N/NH_4_^+^-N ratio was 2.3 for RWS, 31.9 for RW and 147.3 for GB. The low ammonia concentration and the low pH in the GB medium may explain the absence of hairy roots^33^ and potentially shaped the microbial community composition.

Our methodology provided a comprehensive insight into the complex bacterial interactions in horticultural media, supporting our hypothesis that there are fundamental differences between the bacterial communities associated with each type of horticultural growing medium. Diverse and competitive microbial communities may provide different and unique functionalities. As a consequence, the bacterial community inhabiting the GB medium may have provided functional diversity and temporal stability and resilience to this heterogeneous and fluctuating environment. Ultimately, the interactions in the resident community may also play a role in the resistance to external forces, such as invasive species competing in conventional soilless culture systems. Future alternative control strategies may involve the evaluation of the suppressiveness of microbial groups and the transfer of suppressiveness to conducive soils with 0.1–10% suppressive soil^15^. The described relationships will also contribute to the understanding of the functional microbial ecology associated with the growing media and the interaction between microbial community and plants. Knowledge regarding these relationships could potentially be used to develop sustainable strategies to increase plant productivity and quality.

## Methods

### Experimental setting and growing media

The microbial community associated with the different growing media was monitored in a commercial 8.5 ha greenhouse in The Netherlands (51°59’ Latitude and 4°10′ Longitude), cultivating the eggplant *Solanum melongena* cultivar Jaylo (Rijk Zwaan, The Netherlands), grafted on *Solanum lycopersicum* L. x *Solanum habrochaites* Beaufort (De Ruiter, The Netherlands). The two different horticultural growing media were installed at the same time in the greenhouse and the 48 day-old eggplants were planted on top of the two different growing media on the same day. The organic growing medium (GB, Grow Bag, Peltracom, Belgium) was a mixture of white peat (H2-H4 on the von Post scale^34^ [80% v/v] and coconut fibre [20% v/v]). Slabs of GB and mineral medium (RW, Rock wool, Grotop expert, Grodan, The Netherlands) had the following dimensions: 1.0 m × 0.2 m × 0.085 m and 1.0 m × 0.2 m × 0.075 m, respectively. Both growing media were subjected to identical water and fertilizer treatments during the cultivation period according to standard methods, with standard fertigation solution^21^. Two eggplants per slab were planted. Each plant was trained to 3 stems, aiming at a plant density of 1.7 plants/m^2^ resulting in 5.1 stems/m^2^.

### Sample collection

The greenhouse was divided into several blocks each consisting of 6 rows in a randomized block design. Two contiguous blocks were randomly selected and each block contained either RW or GB medium. The two outer rows of each block were not selected, because of possible interactions with the adjacent rows. The eggplants were growing in slabs placed consecutively with an interspacing of 44 cm. One slab was considered an experimental unit. Five slabs from each block were randomly selected from the 4 inner rows and from the two different growing media (GB and RW). Samples of the different experimental units were collected at three time points during the growing season (June, July and August) and at the start of the experiment. Ten subsamples from each experimental unit were collected, pooled, homogenized and treated as a single sample (Supplementary Fig. 3). At each time point, samples were taken from 5 fixed experimental units of each RW and GB, including root material. Each sample of 200 g was divided into 4 homogenous subsamples of 50 g for further analysis: two subsamples (subsample 1 and 2) were used for chemical analyses, one subsample (subsample 3) was stored at 4 °C and used for isolation and identification of *Agrobacterium* sp. and total CFU, as well as humidity determination, and subsample 4 was immediately stored on dry ice, preserved at −80 °C and used for molecular microbial community analysis. The grower reported previous presence of the hairy roots syndrome, which is caused by the pathogen *Agrobacterium rhizogenes*. Hence, disease incidence of the hairy roots syndrome was followed up by a monthly visual inspection of the glasshouse. The hairy roots syndrome was detected in one RW slab at the first time point in June. Further visual inspection during July and August revealed increased incidence of the hairy roots syndrome in RW medium. Additional samples of RW from 5 additional slabs showing visual symptoms of the hairy roots syndrome were taken (named RWS). However, no hairy roots were visually identified in the GB throughout the whole experimental period (December 2012 and November 2013).

### Physicochemical analysis of the growing medium

The physicochemical characteristics of the different growing media were determined at the start (December 2012) and during the growing season (June, July and August 2013). The chemical analysis was performed as described by Gabriels, *et al.*^35^. The humidity (w/w-%) of the growing media was determined according to Verdonck and Gabriels^36^.

### Determination of the hydraulic properties of the growing medium

The soil water retention curve of the RW and the GB media was established using the sand box apparatus^37^ for pressure potentials between −1 and −10 kPa. For this experiment, 10 replicates of the slab samples were used. The parameters of the van Genuchten equation were estimated and data was fitted^38^.

### Isolation, identification and determination of the *Agrobacterium* sp. and total cell count

Growing medium was analysed within 48 hours of sample collection. Five grams of the fresh growing medium were mixed with 45 ml of 0.85% NaCl^39^ and homogenized for 2 minutes, using a Stomacher80 blender (Stomacher, Seward, Worthing, UK). This suspension was used for the determination of the total cell and *Agrobacterium* sp. count on each medium. For the total cell count, the suspension was plated on R2A agar (Sigma Aldrich, Diegem, Belgium) with cycloheximide (200 mg/l). Agrobacteria colonies were selected and identified following Shams, *et al.*^40^. *A. rhizogenes* was isolated using 2E-Te containing erythritol and 320 mg/l K_2_TeO_3_ with cycloheximide. After 5 days of incubation at 28 °C, colony forming units (CFU) were counted for both R2A and 2E-TE medium. The calculation of the CFU was following the procedures outlined by Sutton^41^, where the detection limit was equal to 1 CFU at the lowest dilution.

### DNA extraction

Total DNA was extracted using physical disruption with the bead beating method from Hernandez-Sanabria, *et al.*^42^. Cells were lysed in a FastPrep-96 homogeniser (MP Biomedicals, Illkirch, France) and DNA was precipitated with cold ethanol and resuspended in 30 μl of TE buffer (10 mM Tris-HCl, 1 mM EDTA [pH 8.0]). Concentration and quality of DNA were measured based on the absorbance at 260 and 280 nm in a Nanodrop ND 1000 spectrophotometer (NanoDrop Technologies, Wilmington, DE, USA).

### Identification of *Agrobacterium* sp. at strain/biovar level

The potential presence of pathogenic *Agrobacterium* sp. strains was analysed by multiplex PCR, targeting the 23 S rRNA gene^43^. Universal forward primer UF and four reverse primers specific for *A. tumefaciens* (biovar 1), *A. rhizogenes* (biovar 2), *A. vitis*, and *A. rubi*, were used. Conditions of the PCR were described elsewhere^43^; the primer pairs UF/B1R,UF/B2R, UF/AvR and UF/ArR were employed to amplify fragments of 184, 1066, 478, and 1006 bp length, respectively^44^. Pathogenic plasmid detection revealed the presence of the *virC* pathogenicity gene located on the rhizogenic (Ri) plasmid^45^; PCR conditions for detection of the *virC* gene followed Kuzmanović, *et al.*^44^. Additional confirmation was performed in randomly selected wild type isolates; colony PCR was applied using the protocol described above.

### Community PCR-DGGE analysis

PCR amplifications of the V3 region (~200 bp) of the 16 S rRNA gene of bacteria were performed with universal bacterial primers as described by Øvreås, *et al.*^46^. PCR products were purified prior to fingerprinting analysis and DGGE was run on 1 × TAE buffer (AppliChem, Darmstadt, Germany) with a 6% polyacrylamide gel with a 30 to 50% linear denaturing gradient, using the Bio-Rad DCode universal mutation detection system (BioRad, Hercules, CA, USA). Running conditions and analysis using BioNumerics software, version 5.1 (Applied Maths, Sint-Martens Latem, Belgium) were reported by Hernandez-Sanabria, *et al.*^42^. New band categories including all the detected bacterial phylotypes on the growing media were created. Frequency of phylotypes exclusively present in samples with hairy roots was determined adapting the methodology of Hernandez-Sanabria, *et al.*^47^, for performing Fisher Exact test in R^48^.

### Illumina library generation

The V5-V6 region of the 16 S rRNA gene was amplified using reported primers^49^. Libraries were prepared by pooling equimolar ratios of amplicons (200 ng of each sample), tagged with a unique barcode^50^. Resulting libraries were sequenced on a MiSeq (Illumina, Hayward, CA,USA) paired and joined, but only forward reads were selected for the final analysis (140 nt). A quality filter program that runs a sliding window of 10% of the read length, and calculates the local average score based on the Phred quality score of the FASTQ file, was used to trim the 3’-ends of the reads that fell below a quality score of 10. Reads with an N character in their sequence, mismatches within the primers and barcodes or more than 8 homopolymers stretches were discarded. Following primer sequences trimming, sequences were separated based on their barcodes. Number of representative phylotypes were generated using the Uclust algorithm on USEARCH^51^ by clustering at 97% similarity (1 mismatch), with a confidence level of at least 80, with Cyanobacteria, Eukaryota, and Archaea lineages removed. Filtered database contained only phylotypes present in at least a) one sample at an abundance higher than 1%, b) in 2% of samples at a relative abundance above 0.1%, and 3) in 5% of the samples at any abundance level^50^. Hence, a total of 475995 reads were obtained. Sequence composition of the dataset was compared using the RDP Classifier tool^52^ and SILVA database^53^. After examining read counts, data were randomly rarefied to a chosen maximum depth of 17135 sequences, using the phyloseq package from R^54^ and rarefaction curves were plotted using the vegan package in R^55^. Relative abundances of the top twelve taxa, with their deepest possible RDP classification up to the family level were determined and plotted as bar charts^56^. If any OTU was not classified up to a family level, the consensus sequence was blasted using the NCBI database and taxonomic classification was obtained. Within each sample, total number of species, Fisher’s diversity, Shannon, Simpson and inverse Simpson indices were calculated to assess the alpha diversity. Pielou’s index was used as indicator of evenness in the community. Differences in alpha diversity and evenness measures among horticultural growing medium were compared using a repeated measures mixed model in SAS (version 9.3, SAS Institute, Cary, USA), with growing medium type as a fixed effect and comparing multiple means using Tukey test. Hence, the differences in the diversity measures could be attributed to either time point, growing medium type or to the interaction of time*growing medium type. Chao and Bray-Curtis indices were used to construct dissimilarity matrices of the communities. Therefore, beta diversity of the community was determined, and nMDS was employed to visualise the differences among samples, using the vegan package in R^55^. Stratified permutational multivariate analysis of variance (PERMANOVA) with 999 permutations was conducted to explore the percentage of variance that could be explained by the differences in beta diversity. ANOVA was applied to uncover whether one of the growing media was more variable than the other^55^. Differences in relative abundances of bacterial families were compared using a repeated measures mixed model in SAS, with the lsmeans adjustment and Bonferroni correction for multiple comparisons.

### Multivariate statistical analysis

Differences in physicochemical characteristics of each horticultural growing medium were compared using a mixed model in SAS. Pearson correlations were used to determine the interactions between the physicochemical characteristics and significance was assumed at *P* < 0.05. Sixteen variables were included in the analysis (humidity, pH, conductivity, nitrate-N, ammonium-N, phosphorus, potassium, calcium, magnesium, sulphate, sodium, chloride, iron, manganese, CFU of *A. rhizogenes* sp. and total bacteria). Multiple Factor Analysis (MFA) was employed to detect how the relative abundances of families contributed to the differences between growing media across time points. In addition, MFA was applied to the whole set of variables to assess the correlations among the physical, chemical and microbiological variables detected in both types of growing medium. Each group of variables was weighted and results were explained in a factor map^57^, where the value of the abundance of each bacterial family (vector) for the corresponding growing medium (factor) was plotted. The function MFA from the FactoMineR package^58^ was performed in R. Bipartite networks were inferred using a pair-wise similarity matrix obtained from the Regularised Canonical Correlation Analysis^59^,^60^. The values in the similarity matrix were computed as the correlation between the relative abundances of bacterial families and the growing medium characteristics projected onto the space spanned by the first components retained in the analysis. Three relevant components were obtained setting a threshold of r ≥ 0.5 and families were disseminated in the plot, in close relation with the variables correlated and with the growing medium where they were more abundant. An additional ordination procedure, Correspondence Analysis (CA), was employed to confirm the relationships among specific bacterial families and the assessed physical and chemical characteristics^47^.

## Additional Information

**How to cite this article**: Grunert, O. *et al.* Mineral and organic growing media have distinct community structure, stability and functionality in soilless culture systems. *Sci. Rep.* **6**, 18837; doi: 10.1038/srep18837 (2016).

## Supplementary Material

Supplementary Information

## Figures and Tables

**Table 1:** Effect of time and growing medium type on relative abundance of bacterial families present in horticultural growing media (n = 12).

Taxonomy	Time point	Growing medium		*P* value	Time effect	Time * growing medium interaction
		GB (Mean % ± SEM)	RW (Mean % ± SEM)			
Acetobacteraceae	1	0.18 ± 0.07	0.00	0.03	NS	NS
	2	0.15 ± 0.07	0.00			
	3	0.13 ± 0.07	0.00			
Actinobacteridae	1	0.78 ± 0.42	0.07 ± 0.42	0.04	NS	NS
	2	1.81 ± 0.42	0.87 ± 0.42			
	3	1.38 ± 0.42	0.28 ± 0.42			
Chitinophagaceae	1	19.47 ± 2.89^a^	4.42 ± 2.89^b^	0.05	NS	NS
	2	7.63 ± 2.89	6.65 ± 2.89			
	3	8.16 ± 2.89	6.63 ± 2.89			
Chromatiaceae	1	0.10 ± 0.14	0.02 ± 0.14	0.01	NS	NS
	2	0.42 ± 0.14	0.00			
	3	0.80 ± 0.14	0.00			
Conexibacteraceae	1	0.30 ± 0.60	0.05 ± 0.60	0.03	0.01	NS
	2	2.11 ± 0.60	0.10 ± 0.60			
	3	1.92 ± 0.60	0.09 ± 0.60			
Cryomorphaceae	1	0.02 ± 0.48	0.84 ± 0.48	0.05	NS	NS
	2	0.02 ± 0.48^a^	1.63 ± 0.48^b^			
	3	0.00	0.52 ± 0.48			
Desulfobacteraceae	1	0.08 ± 0.09	0.005 ± 0.09	0.0001	0.003	0.003
	2	1.18 ± 0.09^a^	0.00^b^			
	3	0.62 ± 0.09^a^	0.00^b^			
Ectothiorhodospiraceae	1	0.19 ± 0.08	0.005 ± 0.08	0.05	NS	NS
	2	0.22 ± 0.08	0.02 ± 0.08			
	3	0.13 ± 0.08	0.00			
Erythrobacteraceae	1	0.26 ± 0.03^a^	0.05 ± 0.03^b^	0.03	NS	NS
	2	0.10 ± 0.03	0.02 ± 0.03			
	3	0.18 ± 0.03	0.06 ± 0.03			
Gemmatimonadaceae	1	1.24 ± 0.19^a^	0.03 ± 0.19^b^	0.0003	NS	NS
	2	1.20 ± 0.19^a^	0.19 ± 0.19^b^			
	3	1.39 ± 0.19^a^	0.03 ± 0.19^b^			
Hahellaceae	1	0.08 ± 0.06	0.00	0.04	NS	NS
	2	0.18 ± 0.06	0.00			
	3	0.16 ± 0.06	0.00			
Haliangiaceae	1	0.09 ± 0.08	0.005 ± 0.08	0.0002	0.001	0.001
	2	0.41 ± 0.08^a^	0.02 ± 0.08^b^			
	3	1.22 ± 0.08^a^	0.00^b^			
Hyphomicrobiaceae	1	5.69 ± 1.59	1.53 ± 1.59	0.01	NS	NS
	2	8.90 ± 1.59	4.47 ± 1.59			
	3	6.33 ± 1.59	1.76 ± 1.59			
Hyphomonadaceae	1	0.46 ± 0.21	0.07 ± 0.21	0.01	NS	NS
	2	0.60 ± 0.21	0.11 ± 0.21			
	3	0.95 ± 0.21^a^	0.04 ± 0.21^b^			
Ignavibacteriaceae	1	0.12 ± 0.04	0.00	0.02	NS	NS
	2	0.06 ± 0.04	0.00			
	3	0.17 ± 0.04	0.01 ± 0.04			
Methylocystaceae	1	0.14 ± 0.08	0.00	0.03	NS	NS
	2	0.20 ± 0.08	0.00			
	3	0.19 ± 0.08	0.00			
Methylophilaceae	1	6.34 ± 1.87	1.55 ± 1.87	0.04	NS	NS
	2	5.97 ± 1.87	4.09 ± 1.87			
	3	7.02 ± 1.87	1.95 ± 1.87			
Microbacteriaceae	1	0.28 ± 0.69	1.43 ± 0.69	0.003	NS	NS
	2	0.67 ± 0.69^a^	4.52 ± 0.69^b^			
	3	1.02 ± 0.69^a^	3.94 ± 0.69^b^			
Opitutaceae	1	2.98 ± 0.71^a^	0.13 ± 0.71^b^	0.003	NS	NS
	2	4.40 ± 0.71^a^	0.79 ± 0.71^b^			
	3	2.36 ± 0.71	0.14 ± 0.71			
Phyllobacteriaceae	1	0.48 ± 0.16	0.005 ± 0.16	0.007	NS	NS
	2	0.76 ± 0.16^a^	0.22 ± 0.16^b^			
	3	0.64 ± 0.16^a^	0.09 ± 0.16^b^			
Prochlorococcaceae	1	0.12 ± 0.11	0.07 ± 0.11	0.04	NS	NS
	2	0.11 ± 0.11	0.00			
	3	0.48 ± 0.11^a^	0.00^b^			
Rhodobacteraceae	1	0.41 ± 0.05^a^	0.12 ± 0.05^b^	0.02	0.04	NS
	2	0.18 ± 0.05	0.18 ± 0.05			
	3	0.16 ± 0.05	0.03 ± 0.05			
Rhodocyclaceae	1	0.29 ± 0.48	0.16 ± 0.48	0.04	0.02	NS
	2	0.69 ± 0.48	0.30 ± 0.48			
	3	3.22 ± 0.48^a^	0.73 ± 0.48^b^			
Sinobacteraceae	1	0.39 ± 0.11	0.02 ± 0.11	0.003	NS	NS
	2	0.46 ± 0.11	0.11 ± 0.11			
	3	0.65 ± 0.11^a^	0.06 ± 0.11^b^			
Sorangiineae	1	1.06 ± 0.63	0.03 ± 0.63	0.0004	0.02	0.02
	2	3.58 ± 0.63^a^	0.03 ± 0.63^b^			
	3	6.18 ± 0.63^a^	0.005 ± 0.63^b^			
Verrucomicrobiaceae	1	1.23 ± 0.40	0.57 ± 0.40	0.02	NS	NS
	2	2.72 ± 0.40^a^	0.98 ± 0.40^b^			
	3	1.17 ± 0.40	0.52 ± 0.40			
Vibrionaceae	1	0.12 ± 0.01	0.16 ± 0.01	NS	0.0002	NS
	2	0.03 ± 0.01	0.00			
	3	0.00	0.00			
Xanthobacteraceae	1	0.75 ± 0.33	0.08 ± 0.33	0.02	NS	NS
	2	1.52 ± 0.33^a^	0.15 ± 0.33^b^			
	3	0.74 ± 0.33	0.09 ± 0.33			

GB, organic growing medium, n = 6. RW, mineral growing medium, n = 6. NS = not significant effect. Different superscripts indicate significantly different means. “0.00” indicates zero relative abundance detected.

**Table 2:** Effect of time and hairy roots presence on relative abundance of bacterial families present in horticultural growing media (n = 10).

Taxonomy	Time point	Growing medium		*P* value	Time effect	Time * hairy roots interaction
		RW (Mean ± SEM)	RWS (Mean ± SEM)			
Hyphomicrobiaceae	1	1.53 ± 0.57	0.00	NS	0.01	NS
	2	4.47 ± 0.57	3.86 ± 0.57			
	3	1.76 ± 0.57	1.68 ± 0.57			
Methylophilaceae	1	1.54 ± 0.96	0.00	0.03	NS	NS
	2	4.09 ± 0.96^a^	0.35 ± 0.96^b^			
	3	1.95 ± 0.96	0.005 ± 0.96			
Planctomycetaceae	1	0.005 ± 0.05	0.00	0.0003	0.0007	0.0002
	2	0.02 ± 0.05^a^	1.06 ± 0.05^b^			
	3	0.07 ± 0.05	0.01 ± 0.05			
Rhizobiaceae	1	2.94 ± 0.85	0.00	0.0001	0.03	0.05
	2	1.02 ± 0.85^a^	7.91 ± 0.85^b^			
	3	2.02 ± 0.8^a^	13.02 ± 0.85^b^			
Verrucomicrobiaceae	1	0.57 ± 0.45	0.00	0.02	0.02	0.2
	2	0.98 ± 0.45^a^	3.96 ± 0.45^b^			
	3	0.52 ± 0.45	0.53 ± 0.45			
Vibrionaceae	1	0.16 ± 0.005	0.00	NS	< 0.0001	NS
	2	0.00	0.01 ± 0.005			
	3	0.00	0.00			

RW, mineral growing medium, n = 6. RWS, mineral growing medium with hairy roots, n = 4. NS = not significant effect. Different superscripts indicate significantly different means. “0.00” indicates zero relative abundance detected.

**Table 3:** Correlations (r) among physical and chemical characteristics in organic growing medium for cultivating eggplants (GB, n = 15).

	pH	Nitrate-N	P	Ca	Mg	Sulphate	Chloride	Fe	Mn	Total CFU
Humidity	−0.944** (2)0.940** (3)					−0.952**(2)				
pH						−0.881** (3)				
Conductivity		0.880** (1) 0.996** (2) 0.989** (3)		−0.876** (3)		0.961** (2) 0.983** (3)				
Nitrate-N			0.978** (2)	−0.886** (3)		0.967** (2) 0.950** (3)				
Ammonium-N	−0.883** (1)	−0.897** (2)				−0.960** (2)				0.883** (3)
P								−0.889** (1)	0.961** (1) 0.975** (2)	
K					−0.953** (2)			−0.945** (2)		
Ca	0.956** (3)									0.893** (1)
Mg	0.920** (3)		−0.929** (1)	0.883** (2) 0.967** (3)				0.938** (2)		
Na					0.938** (3)			0.954** (2)		
Chloride	0.999*** (2)							0.962** (3)		
*Agrobacterium* sp. CFU										0.959** (3)

In parenthesis, the time point when the correlation was observed. ***P < 0.0001, **P < 0.05.

**Table 4:** Correlations (r) among physical and chemical characteristics in mineral growing medium for cultivating eggplants (RW, n = 15).

	pH	Conductivity	Nitrate-N	Ammonium-N	P	K	Ca	Mg	Sulphate	Na
Nitrate-N		0.915** (1)								
P	0.933** (3)	0.894** (2)	0.984** (2)	−0.954** (1)						
K	0.930** (3)	0.921** (2)			0.922** (3)					
Ca	0.894** (1)	0.897** (2)	0.951** (2)	−0.941** (1)	0.998** (1) 0.894** (3)					
Mg	0.939** (1) 0.902** (3)	0.906** (2)		−0.912** (1)	0.912** (1) 0.958** (3)	0.907** (3)	0.925** (1) 0.972** (2)			
Sulphate		0.965** (2)	0.967** (1)			0.921** (2)				
Na		0.955** (2)	0.961** (1)		0.932** (2)		0.934** (2) 0.887** (3)	0.955** (2)	0.996** (1) 0.906** (2) 0.941** (3)	
Fe	0.882** (1)						0.883** (3)	0.906** (2)		0.892** (2)
Mn	0.906** (3)	0.970** (2)	0.922** (2)	−0.884** (2)	0.950** (2)	0.880** (2)	0.955** (2)	0.926** (2)	0.874** (2)	0.927** (2)
*Agrobacterium* sp. CFU	0.891** (2)	−0.945** (1)								
Total CFU		−0.997** (1)		−0.906** (3)					−0.947** (1)	−0.942** (1)

In parenthesis, the time point when the correlation was observed. ***P < 0.0001, **P < 0.05.

**Table 5:** Correlations (r) among physical and chemical characteristics in mineral growing medium for cultivating eggplants, with hairy roots syndrome (RWS, n = 10).

	Humidity	pH	Conductivity	Ammonium-N	P	Ca	Mg	Na	*Agrobacterium* sp. CFU
Conductivity		0.949** (3)							
Nitrogen-N		−0.907** (2)							
Ammonium-N		0.876** (2)							
P		0.964** (3)	0.960** (3)						0.995** (2)
K	0.887** (3)			−0.899** (2)	0.893** (2)				
Ca	−0.952** (2)				0.958** (2)				0.953** (2)
Mg	−0.905** (2)				0.953** (2)	0.990** (2)		0.998*** (2)	0.934** (2)
Sulphate		0.913** (3)	0.912** (2)			0.926** (3)			0.974** (3)
Na	−0.918** (2)				0.955** (2)	0.993** (2)			0.939** (2)
Chloride			0.901** (2)						
Fe		−0.907** (2)		−0.898** (2)					
*Agrobacterium* sp. CFU	−0.883** (2)				0.995** (2)	0.953** (2) 0.963** (3)	0.934** (2)	0.939** (2)	
Total CFU	−0.907** (2)				0.983** (2)	0.991** (2) 0.915** (3)	0.991** (2)	0.994** (2)	0.971** (2)

In parenthesis, the time point when the correlation was observed. ***P < 0.0001, **P < 0.05.
