# Supplementary material for: Mineral and organic growing media have distinct community structure, stability and functionality in soilless culture systems
Source: Sci Rep. 2016 Jan 5;6:18837. doi: 10.1038/srep18837 (PMC4700413; doi:10.1038/srep18837)
Supplement: Supplementary Information [file srep18837-s1.pdf]

Mineral and organic growing media have distinct community structure, stability and functionality in soilless culture systems

Oliver Grunert[1,3,5] Emma Hernandez-Sanabria[1,5], Ramiro Vilchez-Vargas[1], Ruy Jauregui[4], Dietmar H. Pieper[4,] , Maaike Perneel[3], Marie-Christine Van Labeke[2], Dirk Reheul[2], and Nico Boon[1]

[1]Laboratory of Microbial Ecology and Technology (LabMET), Ghent University, Coupure Links 653, B-9000 Gent, Belgium

[2]Department of Plant Production, Ghent University, Coupure Links 653, B-9000 Gent, Belgium

[3]Peltracom NV, Skaldenstraat 7a, B-9042 Ghent-Desteldonk, Belgium

[4]Microbial Interactions and Processes Research Group. Department of Molecular Infection Biology. Helmholtz Centre for Infection Research, Inhoffenstraße 7, D-38124, Braunschweig. Germany.

[5] These authors contributed equally to this work

Supplementary table 1. Effect of time and growing medium type on species richness (total species), diversity (Shannon, Fisher's alpha, Simpson and Inverse Simpson indices), and evenness (Pielou's index) of horticultural growing media (n = 12). RW, mineral growing medium, n = 6. GB, organic growing medium, n = 6. NS = not significant effect. Different superscripts indicate significantly different means.

**Index**	**Time point**	**Growing medium**		***P* value**	**Time effect**	**Time * growing medium type interaction**
		**GB (Mean ± SEM)**	**RW (Mean ± SEM)**			
Total species	**1**	209.5 ± 15.48	169 ± 15.48	0.01	NS	NS
	**2**	222 ± 15.48	182.5 ± 15.48			
	**3**	235 ± 15.48 ^{a}	177 ± 15.48 ^{b}			
Shannon	**1**	4.36 ± 0.25 ^{a}	3.46 ± 0.25^{b}	0.003	NS	NS
	**2**	4.64 ± 0.25 ^{a}	3.95 ± 0.25^{b}			
	**3**	4.62 ± 0.25^{a}	3.24 ± 0.25^{b}			
Fisher	**1**	34.92 ± 2.78	27.16 ± 2.78	0.006	NS	NS
	**2**	37.92 ± 2.78	29.37 ± 2.78			
	**3**	40.35 ± 2.78 ^{a}	27.89 ± 2.78^{b}			
Simpson	**1**	0.98 ± 0.03	0.92 ± 0.03	0.04	NS	NS
	**2**	0.98 ± 0.03	0.96 ± 0.03			
	**3**	0.98 ± 0.03^{a}	0.86 ± 0.03^{b}			
Inverse Simpson	**1**	40.03 ± 4.50 ^{a}	14.10 ± 4.50 ^{b}	<0.0001	0.03	NS
	**2**	62.27 ± 4.50 ^{a}	25.46 ± 4.50 ^{b}			
	**3**	57.54 ± 4.50 ^{a}	9.26 ± 4.50 ^{b}			
Pielou	**1**	0.82 ± 0.04^{a}	0.68 ± 0.04^{b}	0.003	NS	NS
	**2**	0.86 ± 0.04	0.76 ± 0.04			
	**3**	0.85 ± 0.04^{a}	0.63 ± 0.04^{b}			

Supplementary table 2. Effect of time and hairy roots presence on species richness (total species), diversity (Shannon, Fisher's alpha, Simpson and Inverse Simpson indices), and evenness (Pielou's index) of horticultural growing media (n = 10). RW, mineral growing medium, n = 6. RWS, mineral growing medium with hairy roots, n = 4. NS = not significant effect. Different superscripts indicate significantly different means.

**Index**	**Time point**	**Growing medium**		***P* value**	**Time effect**	**Time * hairy roots interaction**
		**RW (Mean ± SEM)**	**RWS (Mean ± SEM)**			
Total species	**1**	169 ± 17.39	-	NS	NS	NS
	**2**	182.5 ± 17.39	172 ± 17.39			
	**3**	177 ± 17.39	137.5 ± 17.39			
Shannon	**1**	3.46 ± 0.33	-	NS	NS	NS
	**2**	3.95 ± 0.33	4.16 ± 0.33			
	**3**	3.24 ± 0.33	3.16 ± 0.33			
Fisher	**1**	27.16 ± 3.14	-	NS	NS	NS
	**2**	29.37 ± 3.14	27.42 ± 3.14			
	**3**	27.89 ± 3.14	21.02 ± 3.14			
Simpson	**1**	0.92 ± 0.04	-	NS	NS	NS
	**2**	0.96 ± 0.04	0.98 ± 0.04			
	**3**	0.86 ± 0.04	0.89 ± 0.04			
Inverse Simpson	**1**	14.10 ± 4.51	-	NS	0.01	NS
	**2**	25.46 ± 4.51	40.20 ± 4.51			
	**3**	9.26 ± 4.51	12.06 ± 4.51			
Pielou	**1**	0.68 ± 0.05	-	NS	NS	NS
	**2**	0.76 ± 0.05	0.81 ± 0.05			
	**3**	0.62 ± 0.05	0.64 ± 0.05			

Supplementary Table 3. Correlations between relative bacterial abundances and growing medium across time points, indicated by the Multiple Factor Analysis. Dimensions of the MFA can be described by the categorical variables included in the analysis. For each categorical variable (growing medium and time point), a one-way analysis of variance was performed with the coordinates of the samples on the axis, explained by the time point or growing medium type. Then, for each level of the category (i.e. time point 1, time point 2 or time point 3 or growing medium GB), a Hotelling T^2-test was used to compare the average of the category with the general average (using the constraint P i αi = 0, αi = 0). For instance, the coordinates of the relative abundance of family "x" at GB at time point 1 were compared with the average coordinates of the relative abundance of family "x" in GB. The *P* value associated to this test is transformed to a normal quantile to assess whether the mean of the category is significantly less or greater than 0. Negative values indicate negative correlations.

**Dimension**	**Variance**	**Descriptor**	**Estimate (R^2)**	**P value**	**Taxon**	**Correlation**	***P* value**
					Gemmatimonadaceae	0.903	< 0.0001
					Sinobacteraceae	0.895	< 0.0001
					Sorangiineae	0.852	< 0.0001
					Opitutaceae	0.852	< 0.0001
		Medium	0.909	< 0.0001	Desulfobacteraceae	0.837	0.00005
		GB	6.455	< 0.0001	Actinobacteridae	0.832	0.00006
		RWS	-3.568	0.06	Hahellaceae	0.820	0.00010
1	27.8%	RW	-2.888	0.04	Xanthobacteraceae	0.818	0.00011
					Gaiellaceae	0.814	0.0001
					Oceanospirillaceae	0.805	0.0002
					Hyphomicrobiaceae	0.803	0.0002
					Methylophilaceae	0.801	0.0002
					Phyllobacteriaceae	0.801	0.0002
					Acetobacteraceae	0.801	0.0002

		Methylocystaceae	0.793	0.0002
		Ignavibacteriaceae	0.787	0.0003
		Haliangiaceae	0.780	0.0004
		Chromatiaceae	0.761	0.0006
		Pelobacteraceae	0.760	0.0006
		Conexibacteraceae	0.759	0.0007
		Hyphomonadaceae	0.730	0.001
		Coxiellaceae	0.725	0.001
		Actinomycineae	0.719	0.002
		Ectothiorhodospiraceae	0.718	0.002
		Incertae Rhizobiales	0.712	0.002
		Unclassified Parcubacteria	0.699	0.003
		Acidobacteriaceae	0.677	0.004
		Prochlorococcaceae	0.674	0.004
		Erythrobacteraceae	0.655	0.006
		Geobacteraceae	0.604	0.01
		Rhodobacteraceae	0.570	0.02
		Unclassified Nitrospira	0.549	0.03
		Caedibacter	0.510	0.04
		Micrococcineae	-0.498	0.05
		Rhizobiaceae	-0.499	0.05
		Microbacteriaceae	-0.577	0.02
		Incertae Alteromonadales	0.734	0.001
		Cytophagaceae	0.731	0.001
		Micrococcineae	0.727	0.001
2	13.1%	Family XVII Incertae Sedis	0.721	0.002
		Solimonadaceae	0.721	0.002
		Pseudonocardiaceae	0.705	0.002
		Uncultured Chlorobiales	0.698	0.003

					Acidimicrobineae	0.668	0.005
					Enhygromyxa	0.660	0.005
					Saprospiraceae	0.633	0.008
					Parachlamydiaceae	0.615	0.01
					Bdellovibrionaceae	0.607	0.01
					Kordiimonadaceae	0.537	0.03
					Mycobacteriaceae	-0.544	0.03
					Unclassified Saccharibacteria	-0.577	0.02
					Peptococcaceae	-0.662	0.005
					Xanthomonadaceae	-0.685	0.003
					Pseudonocardineae	0.687	0.003
		Time	0.356	0.06	Propionibacterineae	0.606	0.01
		Medium	0.225	0.19	Bacteroidaceae	0.604	0.01
		RW	1.815	0.12	Comamonadaceae	0.543	0.03
3	10.5%	Tpt 1	1.647	0.18	Incertae.Rhizobiales	0.514	0.04
		Tpt 2	0.839	0.31	Cryomorphaceae	0.502	0.05
		RWS	-1.852	0.12	Geobacteraceae	-0.528	0.04
		Tpt 3	-2.486	0.02	Rhodocyclaceae	-0.582	0.02
					Streptomycetaceae	-0.638	0.008
					Gallionellaceae	0.760	0.0006
					Nitrosomonadaceae	0.671	0.004
					Cryomorphaceae	0.620	0.01
		Medium	0.201	0.23	Propionibacterineae	0.566	0.02
4	9.2%	RW	1.609	0.14	Unclassified Saccharibacteria	0.542	0.03
		RWS	-1.633	0.15	Solirubrobacteraceae	0.536	0.03
					Unclassified Microgenomates	0.513	0.04
					Unclassified Nitrospira	-0.516	0.04
					Caedibacter	-0.532	0.03
5	7.9%				Verrucomicrobiaceae	0.653	0.006

			Planctomycetaceae	0.620	0.01
Time	0.717	0.0003	Bradyrhizobiaceae	0.604	0.01
Medium	0.229	0.18	Simkaniaceae	0.576	0.02
Tpt 2	2.968	0.0003	Piscirickettsiaceae	0.562	0.02
RWS	1.904	0.06	Caldilineaceae	0.560	0.02
Tpt 3	-0.411	0.42	Solirubrobacteraceae	0.558	0.02
RW	-1.176	0.30	Nocardioidaceae	0.532	0.03
Tpt 1	-2.558	0.01	Bacteroidaceae	0.520	0.04
			Vibrionaceae	-0.555	0.03

Supplementary table 4. Effect of time on physical and chemical characteristics of organic and mineral growing media for cultivating eggplants, without hairy roots (n = 30). RW, mineral growing medium (n = 15). GB, organic growing medium (n = 15). NS= no significant effect.

**Variable**	**Growing medium**		***P* value**	**Time effect**	**Time*Growing medium interaction**
	**RW (Mean ± SEM)**	**GB (Mean ± SEM)**			
**Humidity (%)**	85.31 ± 1.03	81.93 ± 1.03	0.05	NS	NS
**pH**	6.58 ± 0.15	6.36 ± 0.15	NS	<0.0001	0.02
**Conductivity (μS/cm)**	545.87 ± 73.38	845.47 ± 73.38	0.02	NS	NS
**Nitrate-N (mg/l)**	263.29 ± 43.63	458.18 ± 43.63	0.01	0.009	NS
**Ammonium-N (mg N/l)**	8.24 ± 0.75	3.11 ± 0.75	0.001	0.01	NS
**P (mg/l)**	86.97 ± 17.87	33.79 ± 17.87	NS	0.05	NS
**K (mg/l)**	315.15 ± 23.24	100.50 ± 23.24	0.0002	NS	0.03
**Ca (mg/l)**	276.17 ± 76.00	1 558.17 ± 76.00	<0.0001	NS	NS
**Mg (mg/l)**	73.17 ± 25.47	524.33 ± 25.47	<0.0001	NS	NS
**Sulphate (mg/l)**	248.53 ± 67.50	737.80 ± 67.50	0.0009	NS	NS
**Na (mg/l)**	56.17 ± 7.82	147.17 ± 7.82	<0.0001	0.05	NS
**Chloride (mg/l)**	10.63 ± 0.94	9.62 ± 0.94	NS	NS	0.0002
**Fe (mg/l)**	4.45 ± 0.32	1.58 ± 0.32	0.0002	0.002	NS
**Mn (mg/l)**	2.63 ± 0.45	1.46 ± 0.45	0.02	NS	NS
***Agrobacterium* sp. (CFU/ml)**	29 473 ± 11 514	7 618 ± 11 514	NS	NS	NS
**Total bacteria (CFU/ml)**	10 682 474 ± 2 609 243	6 722 636 ± 2 609 243	NS	0.0004	NS

Supplementary table 5. Effect of time on physical and chemical characteristics of mineral growing medium with and without hairy roots (n = 25). RW, mineral medium without hairy roots (n = 15). RWS, mineral medium with hairy roots (n = 10). NS= no significant effect.

**Variable**	**Mineral growing medium**		***P* value**	**Time effect**	**Time*Hairy roots interaction**
	**No hairy roots (Mean ± SEM)**	**Hairy roots (Mean ± SEM)**			
**Humidity (%)**	85.31 ± 0.93	83.12 ± 1.11	NS	NS	NS
**pH**	6.58 ± 0.19	7.43 ± 0.24	0.02	0.008	NS
**Conductivity (µS/cm)**	545.87 ± 71.72	815.00 ± 74.65	0.03	NS	0.0003
**Nitrate-N (mg/l)**	263.29 ± 17.67	219.04 ± 38.62	NS	0.0006	0.01
**Ammonium-N (mg N/l)**	8.24 ± 0.70	95.70 ± 1.56	<0.0001	0.002	0.001
**P (mg/l)**	86.97± 26.28	128.62 ± 32.80	NS	NS	0.04
**K (mg/l)**	315.15 ± 32.97	541.48 ± 72.87	0.04	NS	NS
**Ca (mg/l)**	276.17 ± 67.41	174.13 ± 79.80	NS	NS	0.05
**Mg (mg/l)**	73.17 ± 11.34	94.74 ± 14.54	NS	NS	NS
**Sulphate (mg/l)**	248.53 ± 47.11	339.13 ± 56.57	NS	0.005	0.03
**Na (mg/l)**	56.17 ± 7.22	129.63 ± 15.28	0.0009	NS	NS
**Chloride (mg/l)**	10.63 ± 3.68	33.64 ± 4.51	0.003	NS	NS
**Fe (mg/l)**	4.45 ± 0.57	7.50 ± 0.79	0.01	NS	NS
**Mn (mg/l)**	2.63 ± 0.59	2.98 ± 0.71	NS	NS	NS
***Agrobacterium* sp. (CFU/ml)**	29 473 ± 271 685	1 456 074 ± 341 008	0.01	0.02	0.01
**Total bacteria (CFU/ml)**	10 682 474 ± 4 635 147	10 568 506 ± 6 170 457	NS	NS	0.005

Supplementary Table 6. Correlations between physical and chemical characteristics and growing medium across time points, indicated by the Multiple Factor Analysis. Dimensions of the MFA can be described by the categorical variables included in the analysis. For each categorical variable (growing medium and time point), a one-way analysis of variance was performed with the coordinates of the samples on the axis, explained by the time point or growing medium type. Then, for each level of the category (i.e. time point 1, time point 2 or time point 3 or growing medium GB), a Hotelling T^2-test was used to compare the average of the category with the general average (using the constraint P i αi = 0, αi = 0). For instance, the coordinates of calcium in GB at time point 1 were compared with the average coordinates of calcium in GB. The *P* value associated to this test is transformed to a normal quantile to assess whether the mean of the category is significantly less or greater than 0. Negative values indicate negative correlations.

**Dimension**	**Variance**	**Descriptor**	**Estimate (R^2)**	***P* value**	**Variable**	**Correlation**	***P* value**
1	29.8%	Medium	0.692	< 0.0001	Ammonia-N	0.812	< 0.0001
		Time	0.108	< 0.0001	*Agrobacterium* sp. CFU	0.774	< 0.0001
		RWS	1.590	< 0.0001	Phosphorous	0.772	< 0.0001
		Tpt 3	0.510	0.116	Chloride	0.756	< 0.0001
		RW	-0.464	0.265	pH	0.748	< 0.0001
		Tpt 1	-0.618	0.056	Potassium	0.655	< 0.0001
		GB	-1.126	0.000	Iron	0.622	< 0.0001
					Total bacteria CFU	0.476	0.002
					Manganese	0.330	0.04
					Calcium	-0.451	0.003
					Magnesium	-0.473	0.002
					Nitrate-N	-0.589	0.00005
2	26.7%	Medium	0.483	< 0.0001	Sulphate	0.818	< 0.0001

		GB	1.08	< 0.0001	Conductivity	0.806	< 0.0001
		RW	-0.93	< 0.0001	Sodium	0.685	< 0.0001
					Magnesium	0.631	< 0.0001
					Calcium	0.607	0.00003
					Nitrate-N	0.578	0.00008
					Agrobacterium sp. CFU	0.413	0.007
					Potassium	-0.406	0.008
					Iron	-0.419	0.006
					Manganese	-0.507	0.0007
					Humidity	-0.673	< 0.0001
3	13.6%	Time	0.475	< 0.0001	pH	0.429	0.005
		Medium	0.064	0.286	Total bacteria CFU	-0.801	< 0.0001
		Tpt 2	0.609	0.005			
		Tpt 3	0.319	0.308			
		RWS	0.193	0.367			
		GB	0.119	0.464			
		RW	-0.312	0.114			
		Tpt 1	-0.928	< 0.0001			
4	10.8%				Humidity	0.661	< 0.0001
		Tpt 1	0.141	0.475781	Conductivity	0.396	0.01
					pH	-0.337	0.03
5	5.9%	Medium	0.184	0.021	Magnesium	0.410	0.008
		Time	0.041	0.456	Calcium	0.397	0.01
		GB	0.253	0.031	Sulphate	0.317	0.04
		Tpt 3	0.131	0.271	*Agrobacterium* sp. CFU	-0.393	0.01
		Tpt 2	-0.139	0.248			
		RW	-0.324	0.008			

Supplementary table 7. CFU and PCR identification of isolates from growing media used for eggplant cultivation. GB = organic growing medium (n = 15), RW = mineral growing medium (n = 15), RWS = mineral growing medium with hairy roots (n = 10).

**Growing medium**	**Hairy roots**	***A. rhizogenes* bv2 (1066 bp)**	***virC* gene**	***A. rhizogenes* CFU/ml**	***P* value**	**Total bacteria CFU/ml**	***P* value**
GB	0 %	0 %	0 %	7 618 ± 200 726[b]		6 722 636 ± 3 950 556 [a]	
RW	0 %	46.7 %	0 %	29 473 ± 200 726[b]	0.004	10 682 474 ± 3 950 556 [a]	0.19
RWS	100 %	90 %	50 %	1 006 296 ± 234 397[a]		17 940 649 ± 4 613 255 [a]	

*Means with the same letter are not significantly different.

Supplementary Figure 1. Clustering based on the physical and chemical characteristics of the growing media are consistent with hierarchical clustering of bacterial profiles. UPGMA was applied to analyse the PCR-DGGE fingerprints. RW: mineral growing medium; GB: organic growing medium, RWS: mineral growing medium with hairy roots. Tpt, time point.

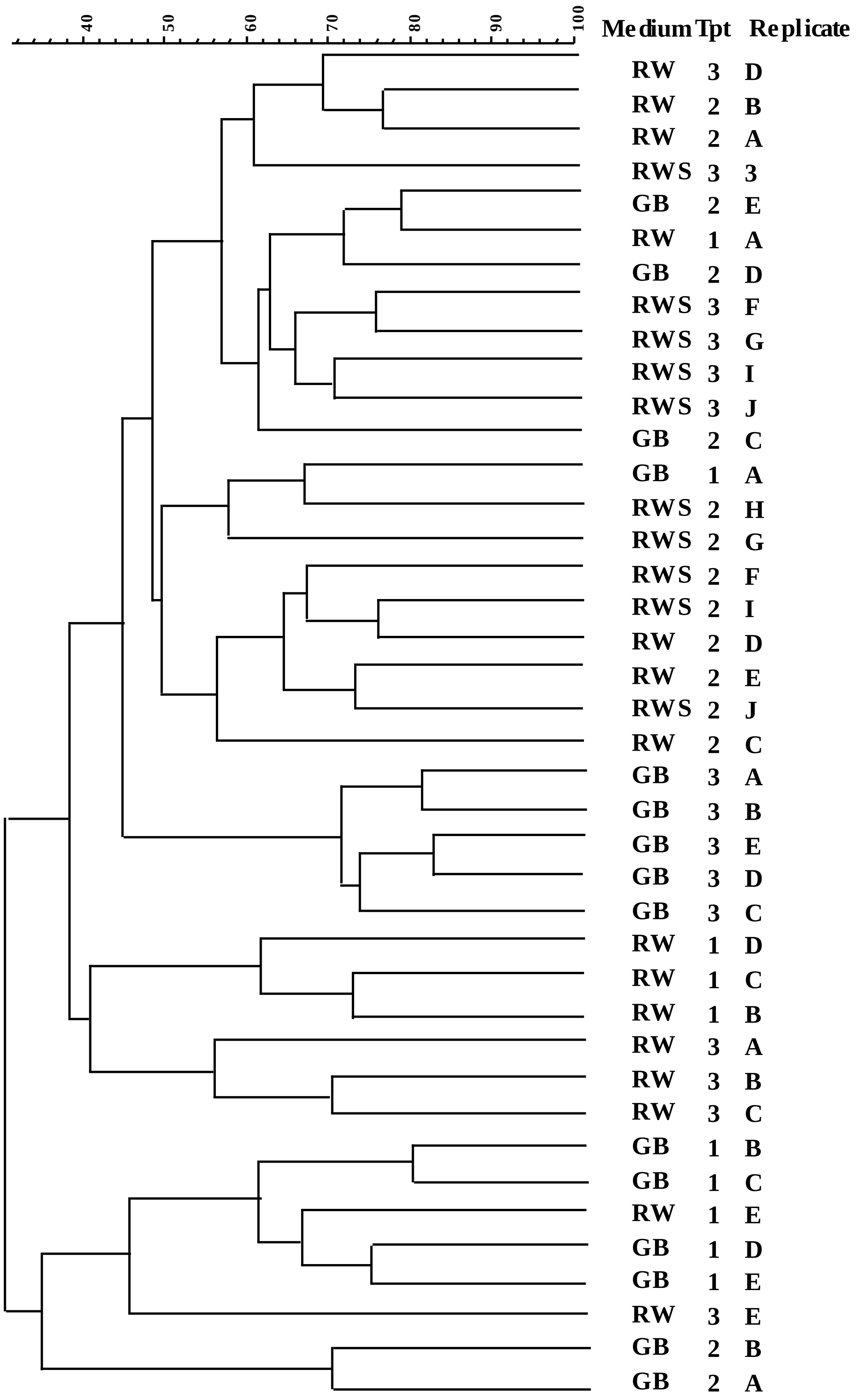

Supplementary Figure 2. Correspondence analysis displaying the relationships among bacterial fingerprints, bacterial relative abundances and physical and chemical characteristics of the horticultural growing media. RW: mineral growing medium; GB: organic growing medium, RWS: mineral growing medium with hairy roots. Symbols indicate the growing medium type: black circles for GB, grey triangles for RW and white squares for RWS. The number in the legend specifies the time point and the letter refers to the sample replicate. For instance, the circle labelled as “1A” refers to the replicate “A” of GB, collected at the first time point.

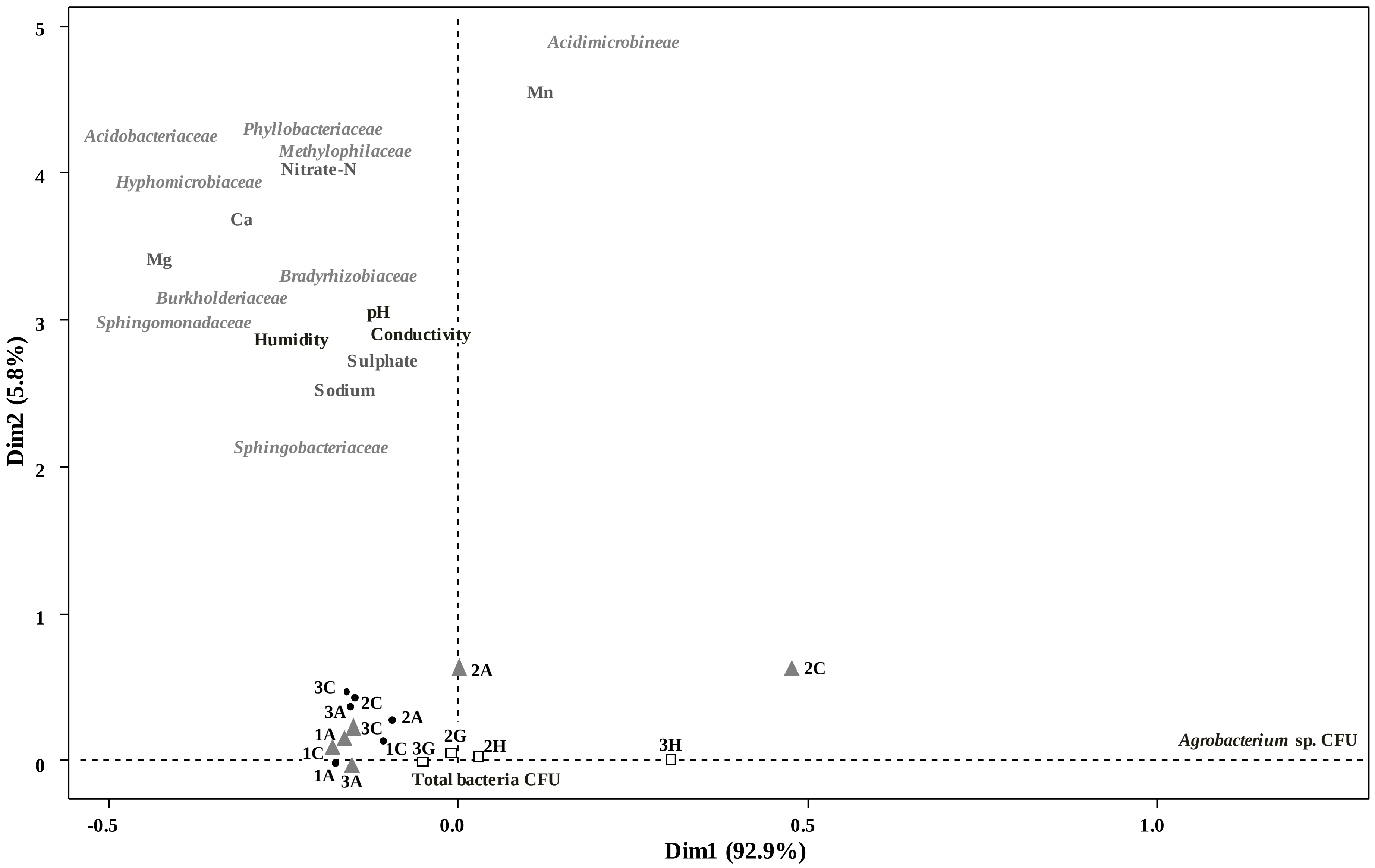
Acidimicrobineae
Mn
Acidobacteriaceae
Phyllobacteriaceae
Methylophilaceae
Nitrate-N
Hyphomicrobiaceae
Ca
Mg
Bradyrhizobiaceae
Burkholderiaceae
pH
Sphingomonadaceae
Conductivity
Humidity
Sulphate
Sodium
Sphingobacteriaceae
2A
2C
3C
2C
3A
2A
1A
3C
1C
3G
2G
2H
3H
Agrobacterium sp. CFU
1C
1A
3A
Total bacteria CFU
Dim2 (5.8%)
Dim1 (92.9%)
-0.5
0.0
0.5
1.0
0
1
2
3
4
5

Supplementary Figure 3. Sample collection and analysis procedure. Two different horticultural growing media were selected for investigating their differences in bacterial communities. One type was constituted by organic material (GB) while other was made of mineral fibres (RW). One slab was considered an experimental unit. Incidentally, plants on RW got naturally infected with the hairy roots (caused by *Agrobacterium* sp.) and those slabs were also sampled (RWS). Ten subsamples were collected from each slab, pooled, homogenized and treated as a single sample (200 ml in total). Samples were taken from 5 fixed slabs of each RW and GB (A, B, C, D and E). Each 200 ml sample was divided into four subsamples: two were used for chemical analyses, one for isolation and identification of *Agrobacterium* sp. and total CFU, as well as humidity determination, and subsample 4 was used for microbial community analysis. Therefore, five samples of GB were stored at times 1, 2 and 3 (n = 15). None were from sick plants. Five samples of RW without hairy roots at times 1, 2 and 3 were collected (n = 15). Plants in RW started to show hairy roots at the time point 2. Therefore, five samples of RWS (F, G, H, I and J) were obtained at time points 2 and 3 (n= 10). Hence, forty samples were used for determination of physical and chemical characteristics. Illumina sequencing was performed in randomly selected, representative samples as follows: from GB and RW, two samples from each time point ($GB_{sequenced}$ = 6, $RW_{sequenced}$ = 6) and two from plants with hairy roots (RWS) at the second and third time points ($RWS_{sequenced}$ = 4).

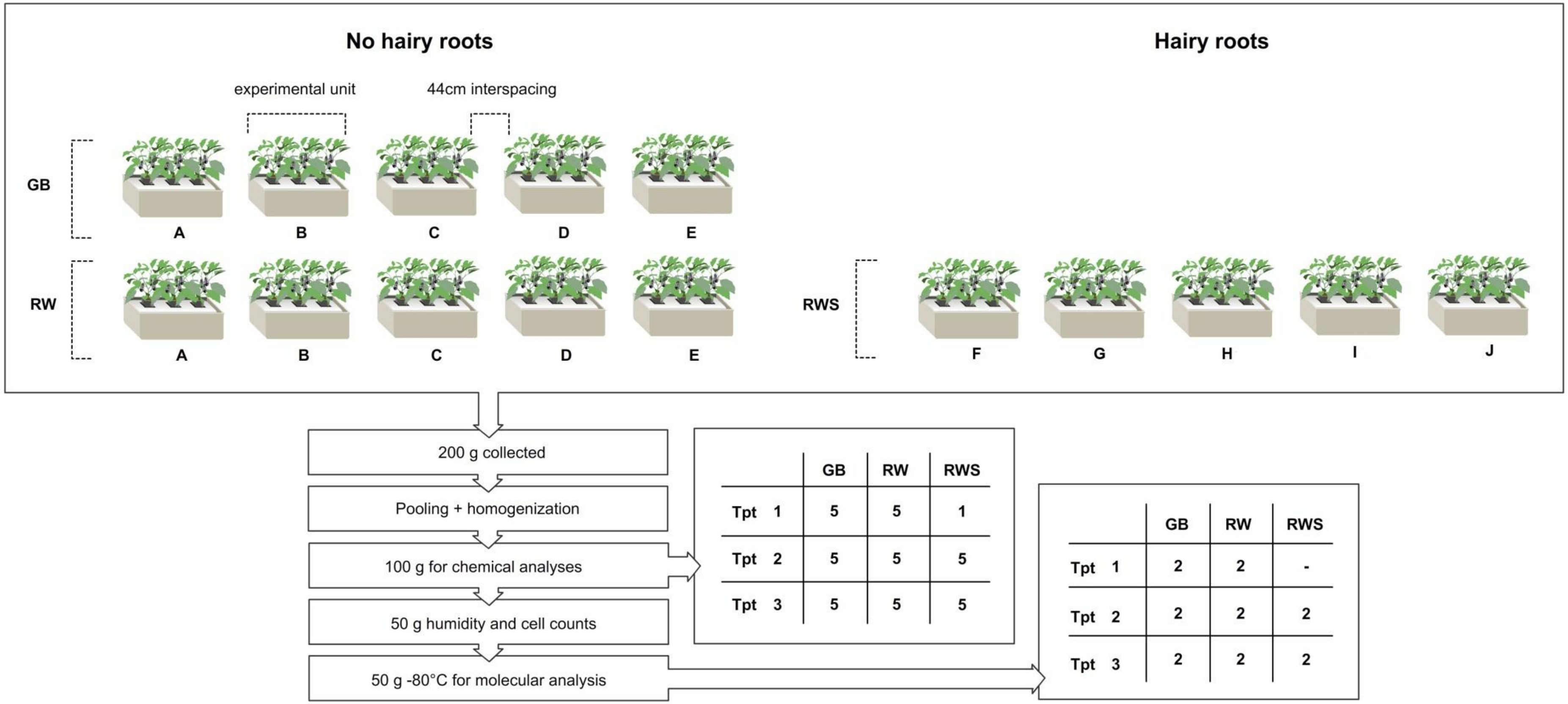
No hairy roots
Hairy roots
experimental unit
44cm interspacing
GB
RW
RWS
A
B
C
D
E
F
G
H
I
J
200 g collected
Pooling + homogenization
100 g for chemical analyses
50 g humidity and cell counts
50 g -80°C for molecular analysis
GB RW RWS
Tpt 1 5 5 1
Tpt 2 5 5 5
Tpt 3 5 5 5
GB RW RWS
Tpt 1 2 2 -
Tpt 2 2 2 2
Tpt 3 2 2 2
